# Modeling Genetic Risk of β‐Cell Dysfunction in Human Induced Pluripotent Stem Cells From Patients Carrying the *MTNR1B* Risk Variant

**DOI:** 10.1111/jpi.70073

**Published:** 2025-09-02

**Authors:** Tania Singh, Sebastian Kalamajski, Joãp. P. M. C. M. Cunha, Siarhei Hladkou, Fiona Roberts, Sevda Gheibi, Anahita Soltanian, Kaveh Yektay Farahmand, Ola Ekström, Anant Mamidi, Paul. W. Franks, Anders Rosengren, Henrik Semb, Hindrik Mulder, Malin Fex

**Affiliations:** ^1^ Unit of Molecular Metabolism Lund University Diabetes Centre Lund Sweden; ^2^ Genetic & Molecular Epidemiology Unit Lund University Diabetes Centre Helsingborg Sweden; ^3^ Translational Muscle Research Unit Lund University Diabetes Centre Malmö Sweden; ^4^ Institute of Translational Stem Cell Research Helmholtz Diabetes Center, Helmholtz Zentrum München Munich Germany; ^5^ Department of Neuroscience and Physiology University of Gothenburg Gothenburg Sweden

**Keywords:** melatonin receptor 1B, single‐nucleotide polymorphism, stem cell derived β‐cells, β‐cell function

## Abstract

Disruptions in circadian rhythm, partly controlled by the hormone melatonin, increase the risk of type 2 diabetes (T2D). Accordingly, a variant of the gene encoding the melatonin receptor 1B (*MTNR1B*) is robustly associated with increased risk of T2D. This single‐nucleotide polymorphism (SNP; rs10830963; G‐allele) is an expression quantitative trait locus (eQTL) in human pancreatic islets, conferring increased expression of *MTNR1B*, which is thought to perturb pancreatic β‐cell function. To understand this pathogenic mechanism in detail, we utilized human induced pluripotent stem cells (hiPSC), derived from individuals with T2D carrying the *MTNR1B* G‐allele. Patient‐derived fibroblasts were reprogrammed to hiPSC and single‐base genome editing by CRISPR/Cas9 was employed to create isogenic lines of either the C/C or G/G genotypes (nonrisk and risk, respectively). In addition, the human embryonic stem cell (hESC) line (HUES4) was subjected to genome editing to create isogenic lines of either the C/C or G/G genotypes. hiPSC and hESC were differentiated into β‐like cells, using a 50‐day 2D protocol. Single‐base genome editing generated cells with the desired genotype at a success rate of > 90%. Expression of stage‐specific markers confirmed differentiation of both hiPSC and hESC into β‐cells. *MTNR1B* mRNA levels were consistently low in differentiated β‐cells, precluding quantitative analysis of gene expression. Western blot analyses indicated slightly higher levels of the MTNR1B protein in differentiated β‐cells carrying the risk allele, which is in accord with the notion that rs10830963 (G‐allele) functions as an eQTL in β‐cells. Insulin secretion in response to the combination of high glucose and IBMX was comparable between genotypes, whereas the addition of melatonin appeared to reduce insulin secretion more efficiently in cells carrying the G‐allele. While our data suggest elevated MTNR1B protein levels in stem cell‐derived β‐like cells carrying the risk allele, these cells do not appear to be sufficiently mature to establish rs10830963 as an eQTL at the mRNA level. The observed nominal increase in melatonin sensitivity in G‐allele–carrying cells is suggestive of a functional contribution of rs10830963 to β‐cell dysfunction; however, this interpretation remains tentative and will require further validation in more mature β‐cell models.

## 1 Introduction

Pancreatic β‐cells reside in small clusters of endocrine cells, termed the islets of Langerhans, which are dispersed throughout the exocrine parenchyma. The β‐cells secrete insulin, a hormone with glucose lowering effects, in response to nutrients absorbed after a meal. This maintains blood glucose levels within a narrow physiological range.

Type 2 diabetes (T2D) is a metabolic disease, the prevalence of which continues to increase globally and represents a major cause of morbidity and mortality. β‐cell function (i.e., the capacity to secrete insulin) plays a crucial role in the development of T2D, as failure to compensate for an increased demand for insulin owing to insulin resistance is central to the pathogenesis of the disease. Although both insulin resistance and β‐cell dysfunction are hallmarks of T2D, impaired insulin secretion is the culprit as insulin resistance alone does not result in T2D [1, 2, 3]. This notion has been further corroborated by genome‐wide association studies (GWAS) of T2D and related traits, which have identified more than 1000 robust genetic association signals [4] that mainly map to genes implicated in β‐cell function [5]. One such genetic signal maps to the *MTNR1B* locus; rs10830963, a single‐nucleotide polymorphism (SNP), located in the intron of the *MTNR1B* gene. It is one of the most robustly replicated risk variants for hyperglycemia and diminished insulin secretion related to T2D development. Carriers of the G‐risk allele exhibit decreased capacity to release insulin, increased fasting plasma glucose and a greater risk of developing T2D (or gestational diabetes) as compared to carriers of the nonrisk allele C‐allele [2, 6, 7, 8, 9]. In addition, rs10830963 is an expression quantitative trait locus (eQTL) conferring increased expression of *MTNR1B* mRNA in islets from risk allele carriers [2]. Indeed, the correlation between disruptions in circadian rhythm, partly regulated by melatonin, appears to increase the risk of type 2 diabetes (T2D) [10].

Despite the strong genetic association of the rs10830963 SNP with T2D and related traits, the precise molecular mechanisms underpinning the role of melatonin signaling in the pathogenesis of T2D have not yet been determined. To this end, we have shown that rs10830963 is an eQTL, conferring increased expression of *MTNR1B* mRNA in human islets of Langerhans [2]. Studies in clonal β‐cells (INS‐1) showed that melatonin reduces both insulin secretion and levels of intracellular cAMP [11, 12], whereas *Mt2* (*Mt2* being the equivalent of the human *MTNR1B* gene) knockout mice display an increase in insulin secretion from isolated islets due to loss of *Mt2* signaling [6]. Indeed, the MTNR1B receptor signals via inhibitory G‐proteins (G_i_) [13], when activated by melatonin, thus abrogating insulin secretion by reducing cAMP. In addition, daily administration of melatonin accentuates the repression of insulin secretion in *MTNR1B* G/G risk allele carriers [6]. Bioinformatics and functional analyses support a pathogenic role of rs10830963, where the risk SNP introduces a putative binding site for the transcription factor NEUROD1, which potentially drives the increased expression of *MTNR1B* in β‐cells [14]. While these observations collectively support a pathogenic role of melatonin signaling in T2D, a causal relationship between rs10830963 (G/G vs. C/C at this position in the DNA) is yet to be established.

Here, we describe a stem cell‐based approach to delineate the role of the *MTNR1B* rs10830963 risk allele in β‐cell function. Homozygous individuals carrying the G‐risk allele were identified and induced pluripotent stem cells (hiPSC) were generated from fibroblasts in skin biopsies. Isogenic, homozygous nonrisk C‐allele hiPSC, were created by single‐base genome editing. Additionally, human embryonic stem cells (hESC) were genome‐edited to generate cells homozygous for risk (G) and nonrisk (C) alleles. Subsequently, cells carrying risk‐ and nonrisk alleles were transcriptionally, morphologically, and functionally compared to gain information about a potential causal relationship between rs10830963 in β‐cells and T2D.

## 2 Material and Methods

### 2.1 Ethical Statement

All participants provided informed consent for donating a skin biopsy. The study was approved by the Swedish Ethical Review Authority, with the ethical statement number Dnr 2019‐05157.

### 2.2 Preparation of Human Dermal Fibroblasts From Adult Skin Biopsies

Patients were recruited from “Detailed assessment of T2D” (DIACT), a sub‐group of ANDIS (All new diabetics in Scania, https://www.andis.lu.se/startsida). A 4.0 mm skin punch biopsy was collected, cut into 0.5–1 mm pieces, and placed in a 6‐well plate containing medium (DMEM, 10% FBS, Pen/Strep, Sigma Aldrich, D6429). A cover slip was placed over the biopsy pieces to fix them to the bottom of the plate. Following incubation for 7–10 days at 37°C, 5% CO_2_, a dense outgrowth of fibroblasts appeared which adhered to the cover slip. Fibroblasts from the cover slip were collected, using trypsin, and further expanded in T‐25 tissue culture flasks as described [15]. Cells were stored in liquid nitrogen or were directly subjected to reprogramming into iPSC.

### 2.3 Reprogramming of Dermal Fibroblasts to iPSC

Reprogramming into hiPSCs was performed in a complete xeno‐free culture environment, using nonintegrating, nonmodified RNAs from a highly efficient and robust Stemgent StemRNA 3rd Gen Reprogramming kit (REPROCELL, Cat. No. 00‐0076). This RNA‐based method combines a cocktail of synthetic, nonmodified reprogramming (OCT4, SOX2, KLF4, cMYC, NANOG, and LIN28) and immune evasion mRNAs (E3, K3, B18) with reprogramming‐enhancing mature, double‐stranded microRNAs from the 302/367 cluster. Use of live staining with Stain Alive TRA‐1‐60 antibody enabled verification and selection of pluripotent clones. More detailed information can be accessed from REPROCELL guidelines.

### 2.4 Sequencing of *MTNR1B* Exon 1 and rs10830963 Alleles From iPSC and hESC

DNA was extracted using DNeasy Blood & Tissue Kit (Qiagen, 69506) or QuickExtract DNA Extraction Solution (Lucigen, QE09050). To genotype the rs10830963 locus, a 601 bp genomic region (chr11:92,975,259‐ 92,975,860, genome build hg38) was amplified with the primers 5′‐TCCAAGTAGCAGTCAGAAGC‐3′ and 5′‐CCAAGTGACATCTCAATGAG‐3′. To determine *MTNR1B* knockout efficiency, a genomic region including the CRISPR/Cas9‐targeted exon 1 (chr11:92,969,727‐92,970,084, genome build hg38) was amplified with the primers 5′‐ TGTCAGAGAACGGCTCCT‐3′ and 5′‐ GGAATAGGTTAGAGTGAAGGGAAAG‐3′. AmpliTaq Gold Master Mix 360 (ThermoFisher, 4398881), at recommended cycling settings, (1 cycle 95°C for 10 min, then 40 cycles of 95°C for 15 s, 58°C for 30 s, 72°C for 45 s, then 1 cycle 72°C for 7 min) was used. PCR amplicons were purified using GeneJET PCR Purification Kit (ThermoFisher, K0701) and Sanger‐sequenced using either the forward 5′‐GTAGCAGTCAGAAGCTGTGGTC‐3′ or the reverse 5′‐GCCTTCCAGAGCCTTTGTTCAG‐3′ sequencing primer (for rs10830963 genotyping) or the 5′‐ATAGGTTAGAGTGAAGGGAAAGGG‐3′ sequencing primer (for *MTNR1B* knockout). The knockout efficiency (indel percentage) was determined using an online tool supplied by Synthego (https://ice.editco.bio/).

### 2.5 Genome Editing of rs10830963 Using CRISPR/Cas9 in iPSC and hESC

Cas9 protein Alt‐R v3 (1072544, HDR enhancer, electroporation enhancer 1075916), as well as custom‐made sgRNA and ssDNA were from Integrated DNA Technologies (Coralville, Iowa, USA). For single‐base editing, to target the risk G‐allele and the nonrisk C‐allele, the spacer sequences TACTGGTTCTGGATAGCAGA and TACTGGTTCTGGATAGGAGA, respectively, were used in single guide RNAs. The synthetic ssDNA homology‐directed repair donor template for the G‐to‐C allele editing was 5′‐GCAGAATATTCCCATCAGGAACCTCCCAGGCAGTTACTGGTTCTGGATAGGAGATGGTGTGAATTCTTAGCATCACTGGGGGCCTGGAGGAGGGGCAGCT‐3′, and for the C‐to‐G allele editing 5′‐GCAGAATATTCCCATCAGGAACCTCCCAGGCAGTTACTGGTTCTGGATAGCAGATGGTGTGAATTCTTAGCATCACTGGGGGCCTGGAGGAGGGGCAGCT‐3′. For *MTNR1B* knockout, single guide RNA targeting exon 1 of *MTNR1B* was used, with the spacer sequence 5′‐GTTGCCCACGACGTCCACGG‐3′. To perform the genomic editing, 135 pmol Cas9 protein Alt‐R v3 were incubated with 150 pmol sgRNA for 30 min before adding 400 pmol electroporation enhancer and for single‐base editing only, 200 pmol ssDNA donor template. The reagents were mixed with 1 million cells resuspended in 100 μL human stem cell buffer kit 2 (Lonza, VCA‐1005); the cells were electroporated using the Amaxa nucleofector IIb program B‐016. For single‐base editing, cells were then placed in culture medium supplemented with 30 μM HDR enhancer and incubated at 32°C for 2 days before moving to 37°C. For knockout editing, the cells were incubated directly in 37°C. After 5 days, an aliquot of cells was used to extract the DNA by QuickExtract DNA Extraction Solution (Lucigen, QE09050). To assess the genomic editing efficiency, PCR amplicons were generated and sequenced as described above. Then, cells were seeded at low density, 500 cells per 10 cm dish, to generate single‐cell clones. Clonal DNA was extracted and PCR of DNA sequence comprising rs10830963 was performed as above. PCR products were screened for allele editing using BsaXI restriction enzyme digests (the C allele creates a BsaXI restriction site), and the edited clones' PCR amplicons were Sanger‐sequenced to confirm proper allele editing and absence of indels.

### 2.6 Culture, Maintenance and Freezing of iPSC and hESC

**Figure 1:**
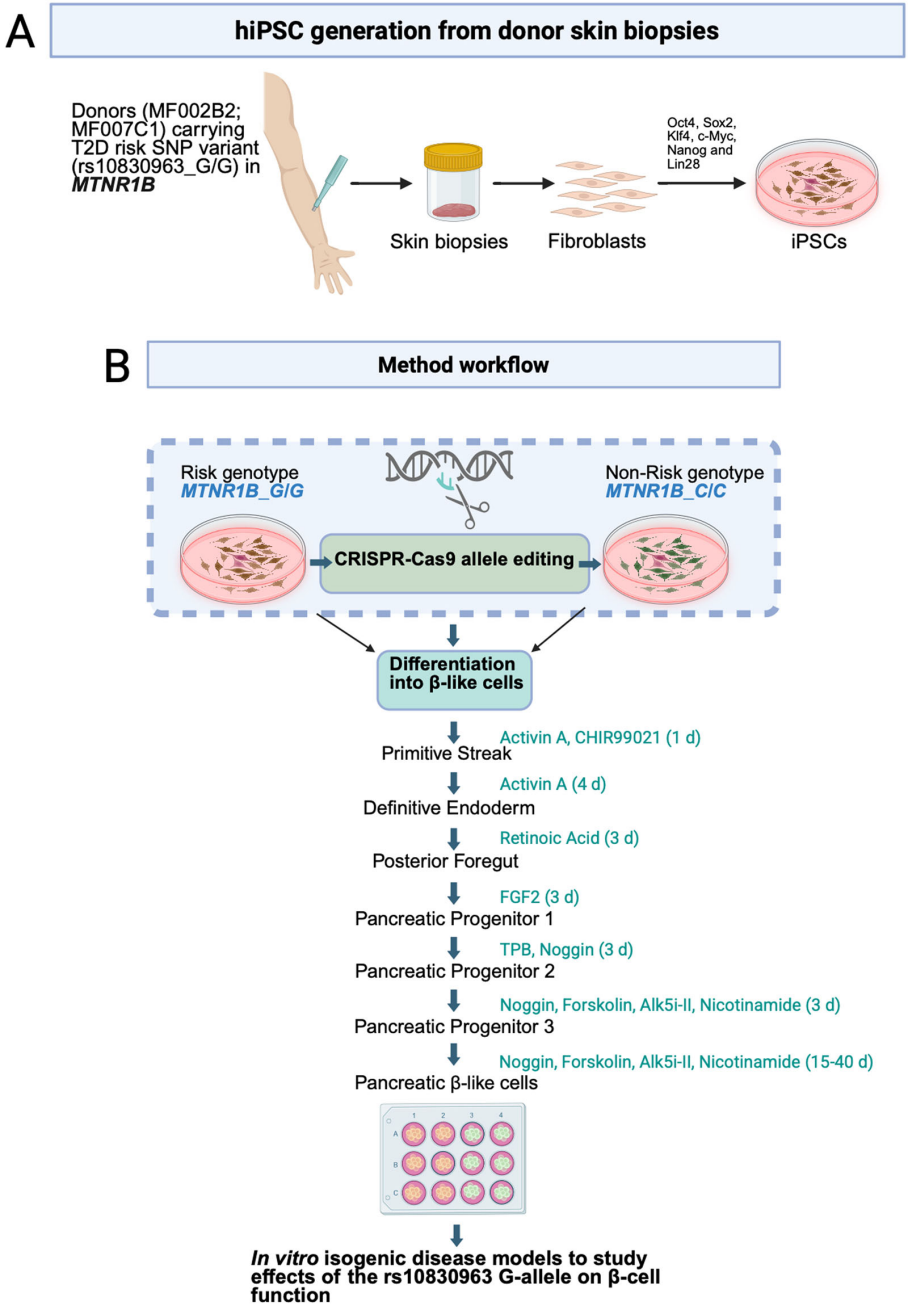
Graphical representation of derivation of hiPSCs and reprogramming into β‐ cells in vitro. (A, B) The image illustrates the workflow of procuring skin biopsies from patients, hiPSC reprogramming, single base genome editing, and differentiation into β‐cells. (B) The procedure creates two isogenic cell lines, which differ only with respect to one single base, that is, the single‐nucleotide polymorphism (SNP) that constitutes the risk allele. Image created in Biorender.

Before differentiation, stem cells were 2D cultured in laminin‐coated (1:20; BioLamina, LN521‐05) T25 flasks with Essential 8 complete medium (for iPSCs, Life Technologies A1517001 and Nutristem Media (for ESCs, Nordic Diagnostica, 05‐100‐1A). Thawing frozen cells required media to be supplemented with RevitaCell supplement (1:100; ThermoFisher, A2644501) for the first 24 h of culture. Upon 80% confluency, cells were washed with DPBS‐/‐ (ThermoFisher, 14040117) and dissociated into single cells using TrypLE select (ThermoFisher, 12563011) for 5 min at 37°C in incubator. Rho kinases inhibitor (10 μM) (Sigma‐Aldrich, Y‐27632A, CAS 146986‐50‐7) was added after every round of passaging for the first 24 h of culture. For freezing of cell stocks, dissociated cells were resuspended in the appropriate volume of PSC cryopreservation medium (Gibco, A2644601) and subsequently stored at −80°C for a day and then transferred to a liquid nitrogen tank for long term storage. Upon defrosting and before culture, hiPSCs generated from fibroblasts and hESCs cell (HUES4) line show robust protein expression of pluripotency markers OCT4 and TRA‐1‐81 (Supporting Information S1: Figure 1).

### 2.7 2D Differentiation of hiPSC/hESC to β‐Cells

Pluripotent cells (⁓100,000 per well) were plated on laminin‐coated (BioLamina, LN521‐05) 12‐well cell culture plates. Differentiation commenced according to a modified protocol [16] and was initiated when cell culture reached ⁓ 95% confluency, and the current stage was designated as differentiation day 0. For Day 0–5, all cells were cultured in RPMI (ThermoFisher, 61870‐044) medium; Day 5–50 cells were cultured in DMEM‐F12 (ThermoFisher, 31331‐093). The cell media (1 mL/well) were supplemented with factors at each developmental stage as follows. Day 0–1, Activin A (100 ng/mL) (Peprotech, 120‐14E), CHIR99021 (3 μM) (Sigma Aldrich, SML1046‐5MG). Day 1–5, Activin A (100 ng/mL) and B27‐insulin (ThermoFisher, A1895601). Day 5–8, Retinoic Acid (2 μM) (Sigma Aldrich, R2625‐50MG). Day 8‐11, FGF2 (64 ng/mL) (Peprotech, 100‐18B). Day 11‐14, TPB (PKC activator; (2S,5S)‐(E,E)‐8‐(5‐(4‐(trifluoromethyl) phenyl)‐2,4‐pentadienoylamino) benzolactam (0.5 μM) (Santa Cruz, 497259‐23‐1) and Noggin (100 ng/mL) (Peprotech, 120‐10C). Day 15‐17, Forskolin (10 μM) (Sigma Aldrich, f3917‐25mg), Alk5i (4.5 μM) (SantaCruz, sc‐221234A), Noggin (100 ng/mL) (Peprotech, 120‐10C), Nicotinamide (10 mM) (Sigma Aldrich, N0636‐100G). Day 17–30 Forskolin (10 μM) (Sigma Aldrich, f3917‐25mg), Alk5i (4.5 μM) (SantaCruz, sc‐221234A), Noggin (100 ng/ml) (Peprotech, 120‐10C), Nicotinamide (10 mM) (Sigma Aldrich, N0636‐100G). In addition, Day 5–50 differentiation media were supplemented with B27 (ThermoFisher, 17504044). Cell culture medium was replaced every 24 h from Days 0–17 and every 48 h from Days 17–50.

### 2.8 Immunoblotting

Cell extracts were prepared using RIPA buffer (Sigma, R0278) supplemented with Pierce Protease Inhibitor Cocktail (ThermoFisher, A32963); the protein concentrations were quantified by BCA protein assay (ThermoFisher, 23225). 20 μg protein were run on a 4%–20% reducing SDS‐PAGE Mini‐PROTEAN TGX gel (BioRad, 4568094), and transferred to a PVDF membrane (BioRad, 1704157) that was blocked for 1 h in 5% BSA (Sigma, A4503) in TBS (Biorad, 170‐6435). Next, the membrane was immunoblotted for 16 h at 4°C in blotting buffer (3% BSA in TBS with 0.1% Tween‐20 (Sigma, P7949) with 1 μg/mL rabbit anti‐MTNR1B (ThermoFisher, PA5‐102107), washed 4 × 5 min in washing buffer (TBS with 0.1% Tween‐20), then incubated for 1 h at room temperature with 0.1 μg/mL HRP‐conjugated goat anti‐rabbit (BioRad, 162‐0177) in blotting buffer, washed 4 × 5 min with washing buffer, then developed using Clarity Western ECL substrate (BioRad, 1705060) and a CCD camera. For reprobing, the membrane was stripped with Restore Western blot analysis Stripping Buffer (ThermoFisher, PIER21059), and the procedure was repeated as above but using 0.2 μg/mL mouse mouse anti‐α‐tubulin (Abcam 7291) as primary, and 0.1 μg/mL HRP‐conjugated goat anti‐mouse antibody (BioRad, 1706516) as secondary antibody. The bands on the immunoblots were quantified using ImageJ software (NIH). The MTNR1B signal was normalized to the β‐tubulin signal.

### 2.9 Immunocytochemistry

hiPSC, hESCs and fully differentiated cells were fixed directly on the culture plates with 4% paraformaldehyde (PFA, Histolab, 2176) for 30 min at room temperature (RT), permeabilized with 0.1% triton X‐100 (Sigma) in PBS (Gibco) for 15 min at RT and blocked with blocking buffer (5% normal donkey serum (NDS) (Abcam, ab7475) + 1% bovine serum albumin (BSA, Sigma, 05482) + 0.1% Tween‐20 (Sigma, P7949) in PBS (Gibco, 18912014) overnight at RT. Primary and secondary antibodies used in this study are described in Supporting Information S1: Table S1. Primary antibody incubation was overnight at 4°C followed by incubation with secondary antibodies in blocking buffer overnight at RT (1:1000, ThermoFisher). Nuclei were stained with 4′,6‐diamidino‐2‐phenylindole, dihydrochloride (DAPI, 1:2000, ThermoFisher, 62248) in 1% BSA in PBS for 10 min at RT. Cells were covered with fluorescence mounting medium (DAKO, S3023) and stored at 4°C. Images were captured by a laser scanning confocal microscope (LSM780, Zeiss). Final images were processed and compiled using Adobe Photoshop and InDesign. Antibodies are described in Supporting Information S1: Table S1.

### 2.10 qRT‐PCR on iPSC and hESC During Differentiation Stages

RNA at the defined stages of differentiation (Day 0, 1, 5, 8, 11, 14, 17, 27, 35, 50) was extracted using RNeasy Plus mini kit (Qiagen, 74136) according to the manufacturer's guidelines. cDNA synthesis with 500 ng total RNA (bulk cells) was performed using RevertAid first strand cDNA synthesis kit (ThermoFisher, K1622) according to the manufacturer's guidelines. Real‐time qPCR reactions were set up in duplicates for each sample, using Taqman assays and Taqman Universal master mix (ThermoFisher, 4305719) in real‐time qPCR system (Applied Biosystems, Quant Studio 7 Flex) using StepOnePlus software. qRT‐PCR data were normalized to the geometric mean of two housekeeping genes (*TBP*, TATA‐box binding protein) and *PPIA* (Cyclophilin A), using the ΔC_T_ method. All TaqMan assays used in this study are listed in Supporting Information S1: Table 2.

### 2.11 pLenti‐HIP‐GFP Virus Production

Lentiviral vectors were generated, as previously described [17], with titers ranging from 10^7^ to 10^8^ TU/mL and determined by fluorescence‐activated sorting (FACS). Briefly, HEK293T cells were cultured to reach a confluency of 80%–90% on the day of transfection. For the production, third‐generation packaging, and envelope vectors (pMDL, psRev, and pMD2G) were used in conjunction with Polyethyleneimine (PEI Polysciences PN 23966) in DPBS (Sigma Aldrich, D8537). The supernatant was then collected, filtered, and centrifuged at 25,000 *g* for 1.5 h at 4°C. The supernatant was removed from the tubes and the virus was resuspended in PBS (Sigma Aldrich, P4474), and left at 4°C. The resulting lentivirus was aliquoted and stored at −80°C. Generated lentivirus was tested and titrated in human EndoC‐βH1 line (Supporting Information S1: Figure S4).

### 2.12 Fluorescence‐Activated Cell Sorting

At Day 45 of differentiation, cells were infected with pLenti‐HIP‐GFP virus (MOI = 2). Five days post‐transduction, robust GFP green fluorescence appeared in β‐cells. For FACS, single cells were prepared using TrypLE select (ThermoFisher, 12563011). All washes were performed in FACS buffer (2% FBS (Sigma Aldrich, A7030‐100G) in DPBS‐/‐ (ThermoFisher, 14040117). Cellular debris were removed as described by the manufacturer's guidelines (Miltenyibiotec, 130‐109‐398). Cells were filtered through 30uM filter (BD Biosciences, 340627) and sorted (BD Aria Fusion) for GFP^+^ cells (Supporting Information S1: Figure S5) in Eppendorf tubes containing FACS buffer. Cells were redistributed in lysis buffer (Norgen Biotech 51800) and stored in −80°C.

### 2.13 qRT‐PCR on Sorted β‐Cells

Sorted, GFP**‐**expressing β‐cells were subsequently processed to obtain RNA, using Single Cell RNA Purification Kit (Norgen Biotech 51800). RNA was quantified by nanodrop spectrophotometer (ND‐1000). cDNA was synthesized from 50 to 80 ng of total RNA according to manufacturer's protocols using SuperScipt IV VILO Mastermix with ezDNASE Enzyme kit (ThermoFisher, 11756050). Real‐time qPCR reactions were set up in duplicates for each sample using Taqman assays and Taqman Fast Advanced master mix (ThermoFisher, 4444556) in real‐time qPCR system (Applied Biosystems, Quant Studio 7 Flex) using StepOnePlus software. qRT‐PCR data were normalized to the geometric mean of two housekeeping genes (*TBP* and *PPIA* using the ΔC_T_ method. All TaqMan assays used in this study are listed in Supporting Information S1: Table S2.

### 2.14 Insulin Secretion

Differentiated β‐cells from hESCs and hiPSCs cultured until Day 50–60 in 12‐well plates, were starved in secretion assay buffer (SAB; 114 mmol/l NaCl, 4.7 mmol/l KCl, 1.2 mmol/l KH_2_PO_4_, 1.16 mmol/l MgSO_4_, 25.5 mmol/l NaHCO_3_, 20 mmol/l HEPES, 2.5 mmol/l CaCl_2_ and 0.2% BSA (fatty acid free), pH 7.2) supplemented with 1 mM glucose at 37°C for 2 h. Following starvation, cells were stimulated for 1 h with SAB containing either low glucose (LG, 1 mM), high glucose (HG, 20 mM) or HG + IBMX (50 μM or 100 μM). Each of the four treatments was performed in the presence or absence of 100 nM melatonin (Sigma, M5250). Supernatant was collected from the wells and centrifuged (4000 × *g*, 5 min, 4°C). To quantitate intracellular insulin and protein, cells were rinsed in the wells with DBPS and lysed with ice‐cold RIPA buffer supplemented with protease/phosphatase inhibitor (ThermoFisher, A32961), scraped from the well bottom and rotated on a shaker (1600 rpm, 30 min, 6°C). Lysates were centrifuged (12 000 × *g*, 15 min, 4°C) and supernatant collected. Insulin was quantified using human insulin ELISA kit (Mercodia, 10‐1113‐10) according to the manufacturer's guidelines. Insulin concentration in the samples was interpolated from the standard curve using cubic spline regression (Prism/GraphPad). Protein concentration was determined with Pierce BCA Protein Assay Kit (ThermoFisher, 23225).

### 2.15 Data Analysis and Interpretation

Statistical testing for two groups was performed using two‐tailed Student's *t*‐test and the Mann–Whitney *U* test was applied when normal distribution was not apparent. A *p* value of < 0.05 was considered statistically significant. Data are presented as mean±SD. Insulin secretion data were calculated as fold secretion of glucose control (low glucose [LG]) and presented as nominal data. Data are presented as mean±SD. All graphs and analyses were undertaken using the Graphpad Prism 10 software.

## 3 Results

### 3.1 CRISPR‐Cas9 Editing of hiPSC and hESC at the *MTNR1B* Locus

**Figure 2:**
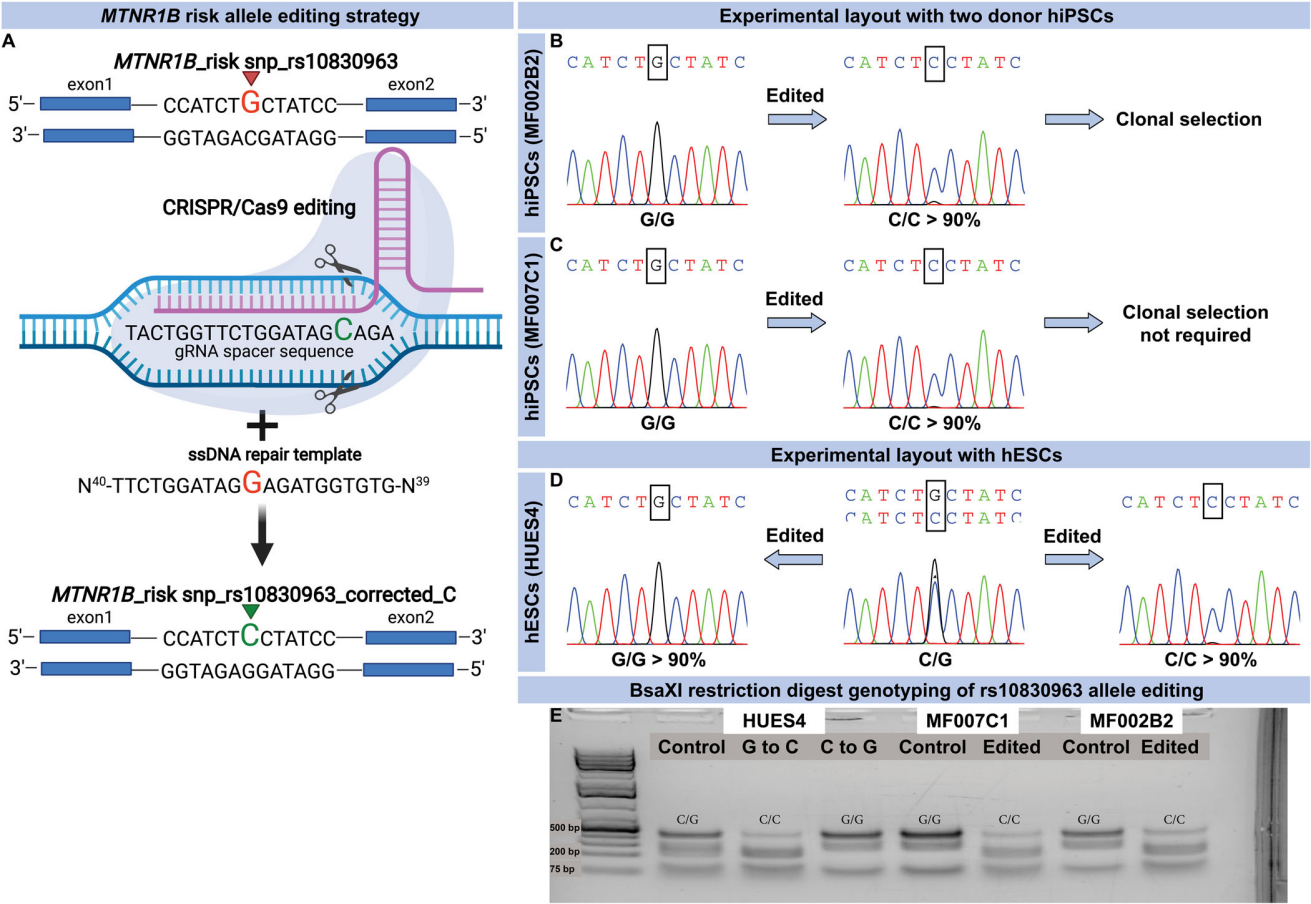
Genome editing of the *MTNR1B* (rs10830963) risk allele and edited clone selection strategy. (A) The graphical representation shows the sequence surrounding rs10830963 in the intron of the *MTNR1B gene*. G, indicated by red arrowhead, is the minor allele conferring the increased risk of impaired insulin secretion and future risk of T2D. A guide RNA (in purple)‐targeting Cas9 to this sequence was prepared and introduced to hiPSCs along with a single strand (SS) DNA template which contains a G instead of a C, thus G will be transcribed to a C (green) instead of a G, resulting in a correction of the G‐risk allele. (B–C) Sanger sequencing of DNA in hiPSCs carrying the homozygous (G/G) risk allele of *MTNR1B* and upon editing; sequencing after single base gene editing in a pool of hiPSC where the vast majority of cells now carries the nonrisk C‐allele. Image created in Biorender (A) A small peak below the major peak indicates that a minor fraction of cells still remains unedited. (B) MF002B2 edited cells were expanded and clonal cell lines were selected as pure edited and control lines. (C) As the editing efficiency was more than 90%, clonal selection for edited MF007C1 cells was not performed. (D) hESCs (HUES4) are normally heterozygous G/C genotype at the rs10830963 locus in *MTNR1B* gene. Therefore, these cells were edited both ways from G to C and C to G in parallel to create cell lines carrying homozygous nonrisk C/C and risk G/G risk alleles. Editing efficiency was more than 90%, hence no clonal cell lines were prepared. (E) Editing was also assessed by BsaXI restriction digestion where G/G risk genotype resulted in a prominent DNA band at a roughly 510 bp size and C/C nonrisk genotype at a roughly 210 bp size.

hiPSC from two human donors (MF002B2 and MF007C1) diagnosed with T2D were subjected to single‐base genome editing of the *MTNR1B* (rs10830963) risk allele (G/G), using CRISPR/Cas9 (See Table 1A–C for patient information and Figure 1A,B for work‐flow of skin biopsy procurements for generation of hiPSC with genome editing and subsequent differentiation to β‐cells). Isogenic hiPSCs were generated carrying nonrisk variants (C/C); editing efficiency was > 90% (Figure 2A–C). In addition, the hESC line, HUES4 [18], was edited at the *MTNR1B* locus (original genotype C/G) to either C/C (nonrisk) or G/G (risk), respectively (Figure 2D), with an efficiency comparable to that in hiPSC clones. Successful editing was confirmed by BsaXI enzyme restriction digestion, where cutting of DNA with G/G risk genotype generated a 510 bp DNA band while DNA with C/C nonrisk genotype generated a 210 bp band (Figure 2E). Before differentiation of hiPSC and hESC to β‐cells, MTNR1B protein was readily detected by immunocytochemistry in all iPSC cultures. MTNR1B protein was observed throughout the cytosolic compartment as well as in the plasma membrane (see enhanced images; Supporting Information S1: Figure S2).

**Table 1:** Donor information, *MTNR1B* genotype and their diabetogenic profile. (A) Table describes the two donors with T2D included in the study and their risk SNP G/G genotype at the *MTNR1B* locus. (B) Shows insulin and (C) glucose measurements upon an oral glucose tolerance test.

1A.				
Donor ID	AGE	BMI	Fasting glucose	MTNR1B genotype rs10830963
MF002	71	27.53	7.60	G/G risk
MF007	63	36.09	9.50	G/G risk

1B.							
Donor ID	Insulin 0 min	Insulin 15 min	Insulin 30 min	Insulin 45 min	Insulin 60 min	Insulin 90 min	Insulin 120 min
MF002	15.0	24.0	32.0	29.0	31.0	50.0	63.0
MF007	26.0	43.0	63.0	69.0	71.0	88.0	104.0

1C.							
Donor ID	Glucose 0 min	Glucose 15 min	Glucose 30 min	Glucose 45 min	Glucose 60 min	Glucose 90 min	Glucose 120 min
MF002	7.5	8.2	9.4	8.8	11.5	14.6	15.5
MF007	9.4	10.1	13.3	15.7	18.5	19.4	19.5

### 3.2 Differentiation of hiPSC and hESC Into β‐Cells

**Figure 3:**
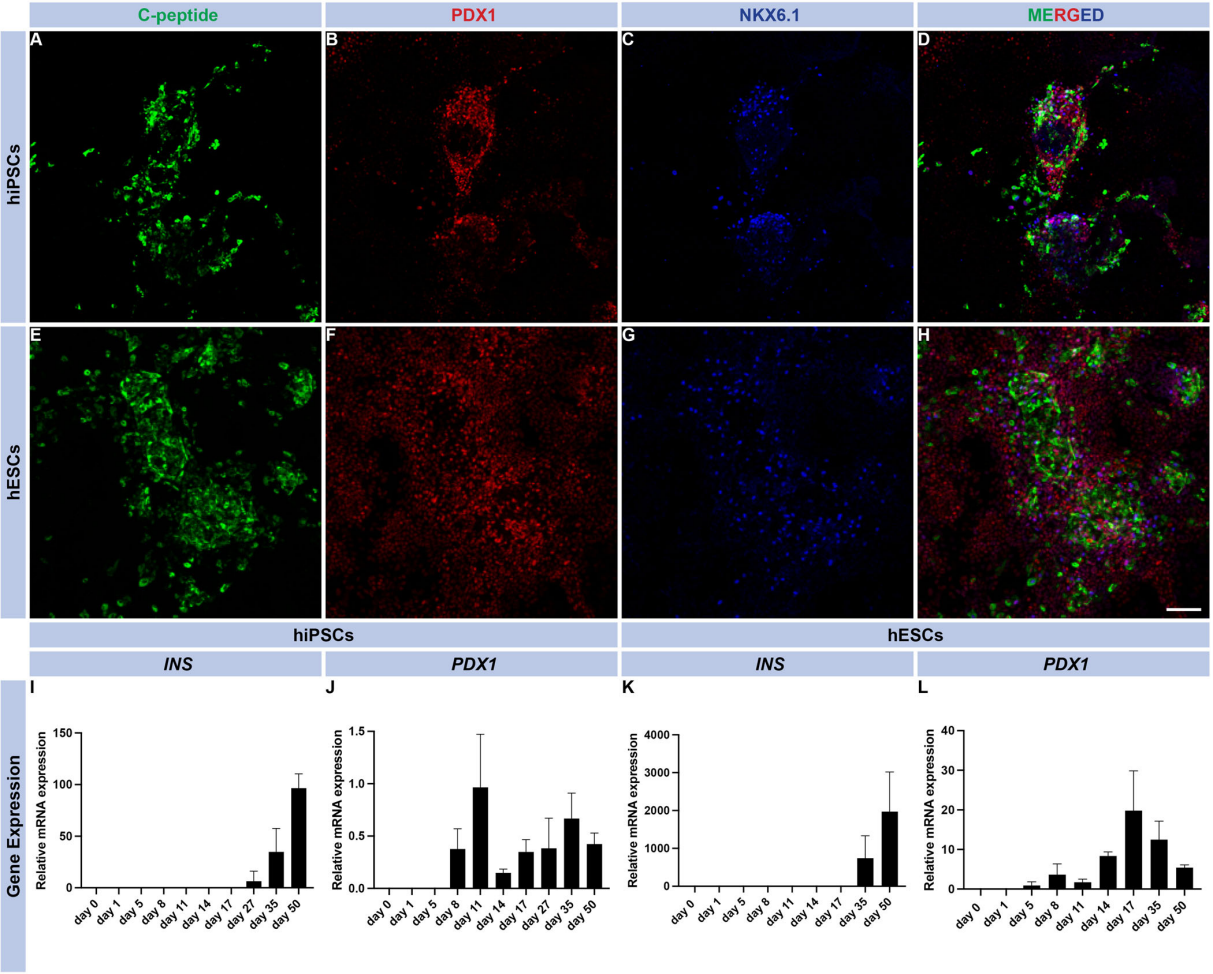
hiPSC and hESC lines and β‐cell differentiation characterization. Immunocytochemistry images of differentiated hiPSCs and hESCs that display C‐peptide^+^ cells (β‐cells in green) co‐expressing PDX1 (red), and NKX6.1 (blue) in fully differentiated cells at Day 50 of differentiation (A–H). The scale bar represents 100 μm, and the images were captured at 20× magnification with a confocal microscope. Gene expression profile of *INS* and *PDX1* in hiPSC and hESC derived β‐cells differentiated for 50 days (I–L) (*N* = 3 biological replicates, mean ± SD).

Successfully edited hiPSC and hESC clones were differentiated into β‐cells employing a protocol for 2D‐culture [16], where addition of small molecules and growth factors enabled pancreatic and islet cell development (Figure 1B). All clones were immunohistochemically evaluated following differentiation, using immunocytochemistry for pancreatic endocrine markers (PDX1 and NKX6.1) as well as C‐peptide during the later stages of differentiation (Figure 3A–D for hiPSCs; E–H for hESCs). Importantly, *INS* (insulin mRNA) was expressed in both hiPSC‐ and hESC‐derived cells from Day 35 (Figure 3I,K). This was also confirmed at the protein level by immunohistochemical staining of hiPSC and hESCs with antibodies to C‐peptide at day 50 of differentiation (Figure 3A,E). Of note, *INS* expression was approximately 20‐fold higher in the hESCs as compared to hiPSCs (Figure 3K vs. 3I), suggesting a higher capacity of hESCs to differentiate into β‐cells. Similarly, *PDX1* expression was observed at day 8 of differentiation in both hiPSC and hESC: its expression varied during the course of differentiation; hESCs expressed *PDX1* at a 10‐fold higher level than hiPSCs at the end of the protocol (Figure 3L vs. 3J). Insulin (C‐peptide staining) was distributed in cells throughout the culture plate (Supporting Figure 3 ‐ tile scans; A and B, hiPSC clones and C, hESCs). Immunohistochemical staining revealed both insulin‐ and glucagon‐positive cells, with some bi‐hormonal cells also observed in the cell cultures (Supporting Information S1: Figure S3D–F). Glucagon (*GCG*) gene expression in hiPSCs and hESCs was detected from Day 35 in hiPSC and hESCs (Supporting Information S1: Figure 3G,H respectively). Importantly, *GCG* gene expression was approximately 18‐fold lower than *INS* gene expression in hiPSC derived endocrine cell cultures and 10‐fold lower in hESC derived endocrine cell cultures.

### 3.3 The *MTNR1B* Risk Variant rs10830963 Is Associated With Altered Protein but Not mRNA Levels of *MTNR1B* in hiPSC Differentiated to β‐Cells

**Figure 4:**
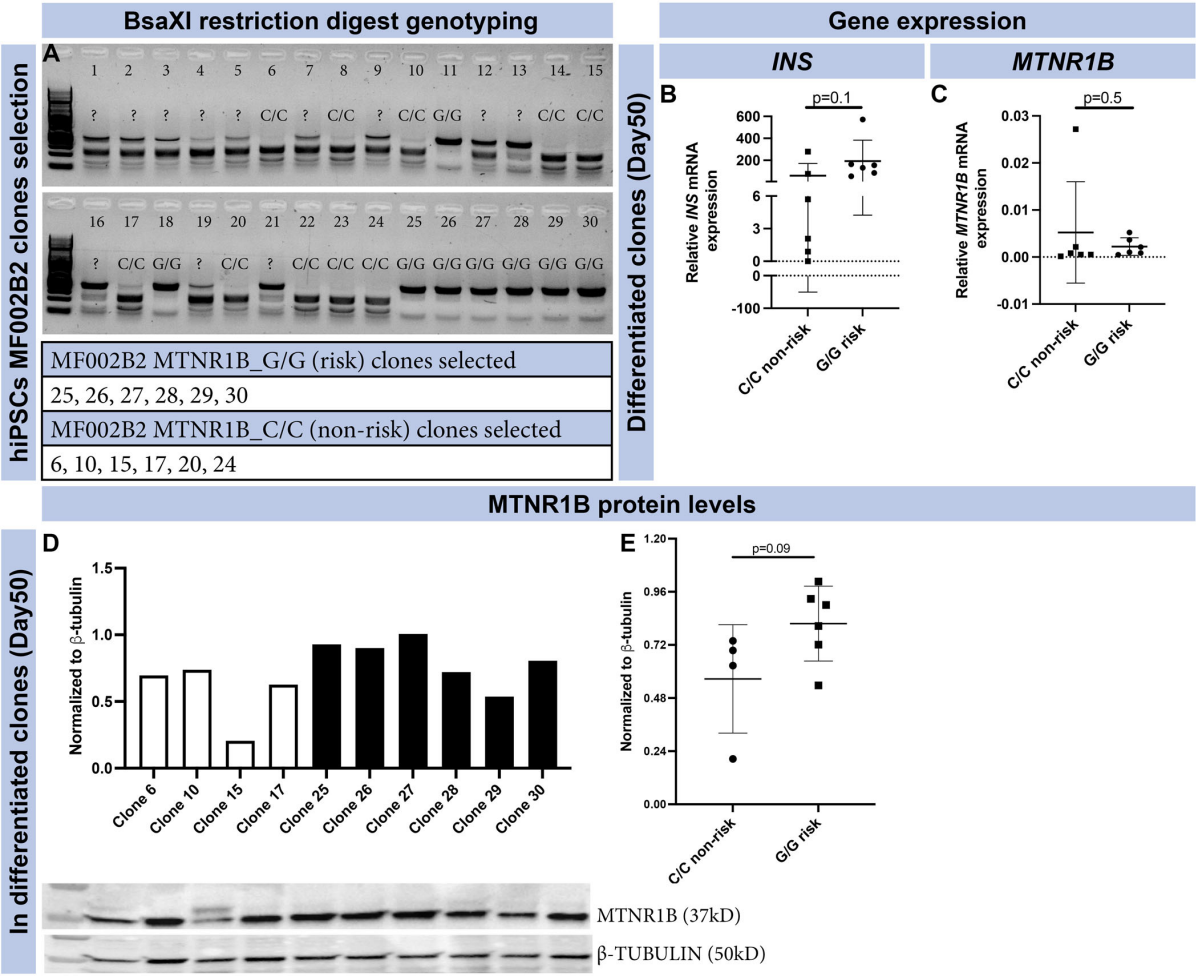
MF002B2 hiPSC line, clonal selection and *MTNR1B* expression. Edited nonrisk (C/C) and risk (G/G) clones were selected based on BsaXI restriction digestion genotyping (A). All clones with clear bands were selected (lower panel in A). At the end of differentiation, cells (bulk) were assessed for gene expression of *INS* (B) and *MTNR1B* (C) (*N* = 6, mean ± SD; each data point represents one individual differentiation experiment from each selected clone). (D, E) MTNR1B protein levels were assessed at the end of differentiation in bulk cells and presented as individual bars for each clone (D: white bars edited C/C clones and black bars, unedited G/G clones) as well as cumulatively (E) (*N* = 4–6, mean ± SD; each data point represents one individual differentiation experiment from each selected clone, (*p* > 0.05 was considered significant).

To determine whether the *MTNR1B* risk and nonrisk variant impacted expression of *MTNR1B*, we used isogenic clones of hiPSC carrying either edited (C/C; nonrisk) and nonedited (G/G; risk) *MTNR1B* alleles (Figure 4A). Six clones of each genotype (C/C and G/G) were chosen, using 2D‐cultures for subsequent differentiation of hiPSC into β‐cells. At Day 50 of differentiation, expression of *INS* and *MTNR1B* was determined by qPCR (Figure 4B,C). No difference in *INS* or *MTNR1B* expression was observed between the genotypes (six clones/genotype from one patient donor). At the protein level, however, western blot analysis demonstrated a trending increase (*p* = 0.09) of MTNR1B protein in β‐cells carrying the G/G risk allele (Figure 4E). To validate the specificity of the antibody used in our western blot, we utilized one of our hiPSC lines to generate an *MTNR1B* knockout line, performing CRISPR/Cas9 mediated deletion of exon 1 in the *MTNR1B* gene. Importantly, this cell line was not clonally selected (i.e., generated from a single cell)—thus, we expexted a mixed cell population of cells maintaining the intact gene and cells containing the knockout. We compared the abundance of the MTNR1B protein in the *MTNR1B* knockout line and isogenic controls by western blot (Supporting Information S1: Figure S6A); we observed a significant reduction of the protein in the knock out line (*p* = 0.007; Supporting Information S1: Figure S6B). Quantification of knockout efficiency of cells was perfomed by Sanger sequencing, The analysis shows 26% knockout‐generating indel mutations ( +1 and −1 base pair) in the cell population after transfection with the denoted guide target (Supporting Information S1: Figure S6C,D). Original blots and ladder are depicted in (Supporting Information S1: Figure S6E–G).

**Figure 5:**
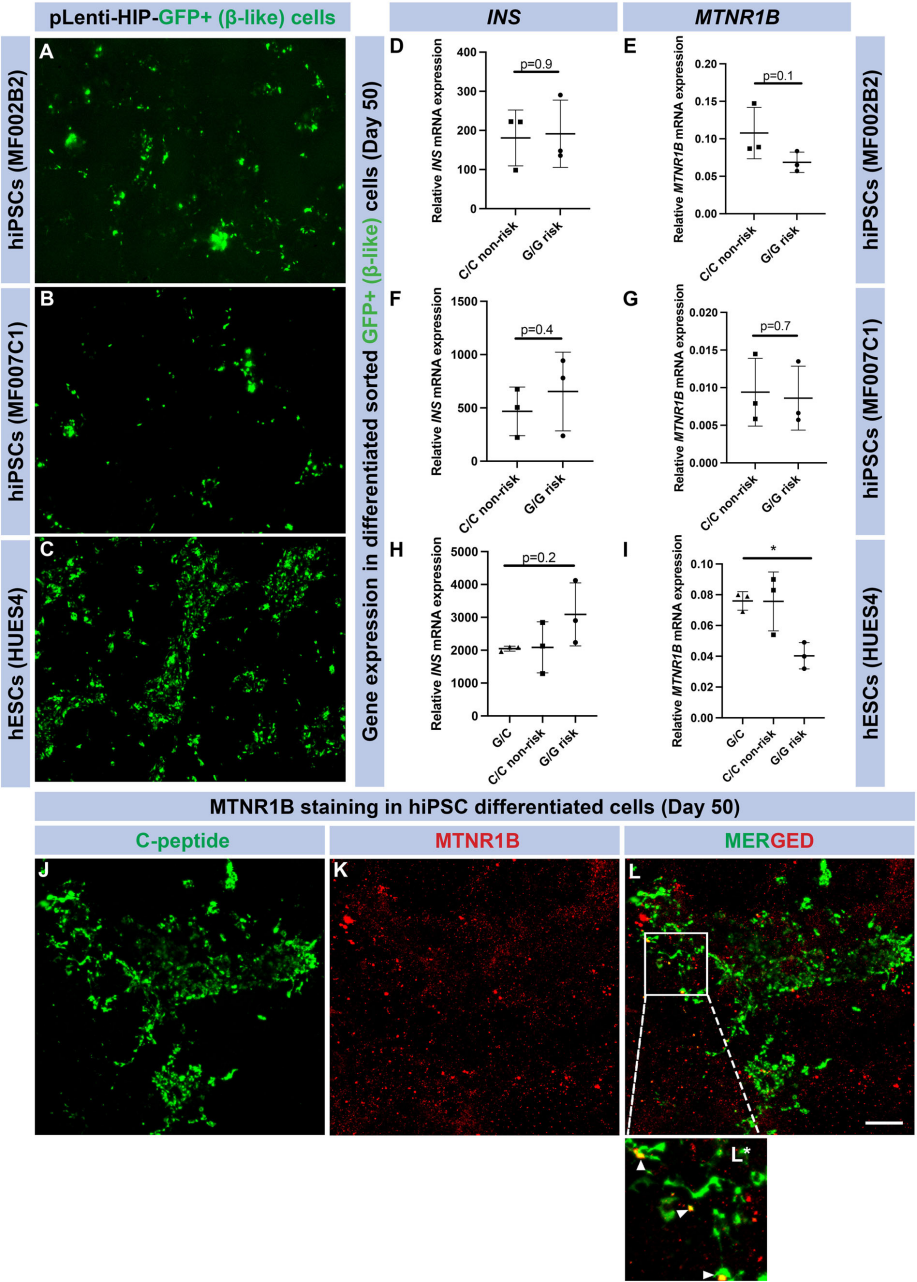
*MTNR1B* expression in fully differentiated β‐cells. Images of GFP expression (green) in hiPSC and hESC derived β‐cells post lentivirus infection (A–C). Graphs show *INS* (D, F, H) and *MTNR1B* (E, G, I) expression in sorted GFP + β‐cells (*N* = 3, mean ± SD; each data point represents one individual differentiation experiment). Immunocytochemistry images show robust C‐Peptide+ cells (β‐cells, green) (J), MTNR1B (red) expression (K), with merge of the two stainings (L) and enhanced image displaying colocalization (L*). Scale bar represents 100 μm, and the images were captured at 10× magnification with a confocal microscope.

To enhance detection of differences in *MTNR1B* expression in β‐cells from isogenic risk and nonrisk allele carriers, we further increased the proportion of β‐cells by FACS of edited and nonedited hiPSC and hESCs. Five days before FACS, cell lines were infected with a pLenti vector expressing green florescent protein (GFP) under the control of the human insulin promoter (HIP; MOI 2; Figure 5A–C). Hereby, insulin‐producing cells would be detected and amenable to sorting by FACS (Supporting Figure 5A–C). Next, we investigated the expression of *INS* and *MTNR1B* in these cell preparations (Figure 5D–I). Again, we were unable to detect any differences in mRNA expression of either *INS* or *MTNR1B* in the β‐cells derived from hiPSC with the G/G and C/C genotypes, respectively. Surprisingly, we observed reduced *MTNR1B* gene expression (*p* = 0.02) in β‐cells derived from hESCs carrying the G/G risk allele compared with hESCs of the C/C genotype (Figure 5I). This is contrary to what we previously have observed in human islets of Langerhans, utilizing qPCR or RNA sequencing respectively [2, 6], where a significant increase in the *MTNR1B* mRNA in nondiabetic G/G risk allele carriers is evident. Regardless of this and of which cell line analyzed, *MTNR1B* expression was consistently very low, just above the level of detection. We then immunostained for C‐peptide and MTNR1B receptor protein in hiPSC derived β‐cells at Day 50 of differentiation (Figure 5J–L). C‐peptide staining was abundant (Figure 5J). MTNR1B staining appeared scattered to several C‐peptide negative cells (Figure 5K,L), as well as appearing in C‐peptide positive cells (Figure 5L merge—including enhanced image (5 L*).

### 3.4 Insulin Secretion From β‐Cells Derived From hiPSC and hESC

**Figure 6:**
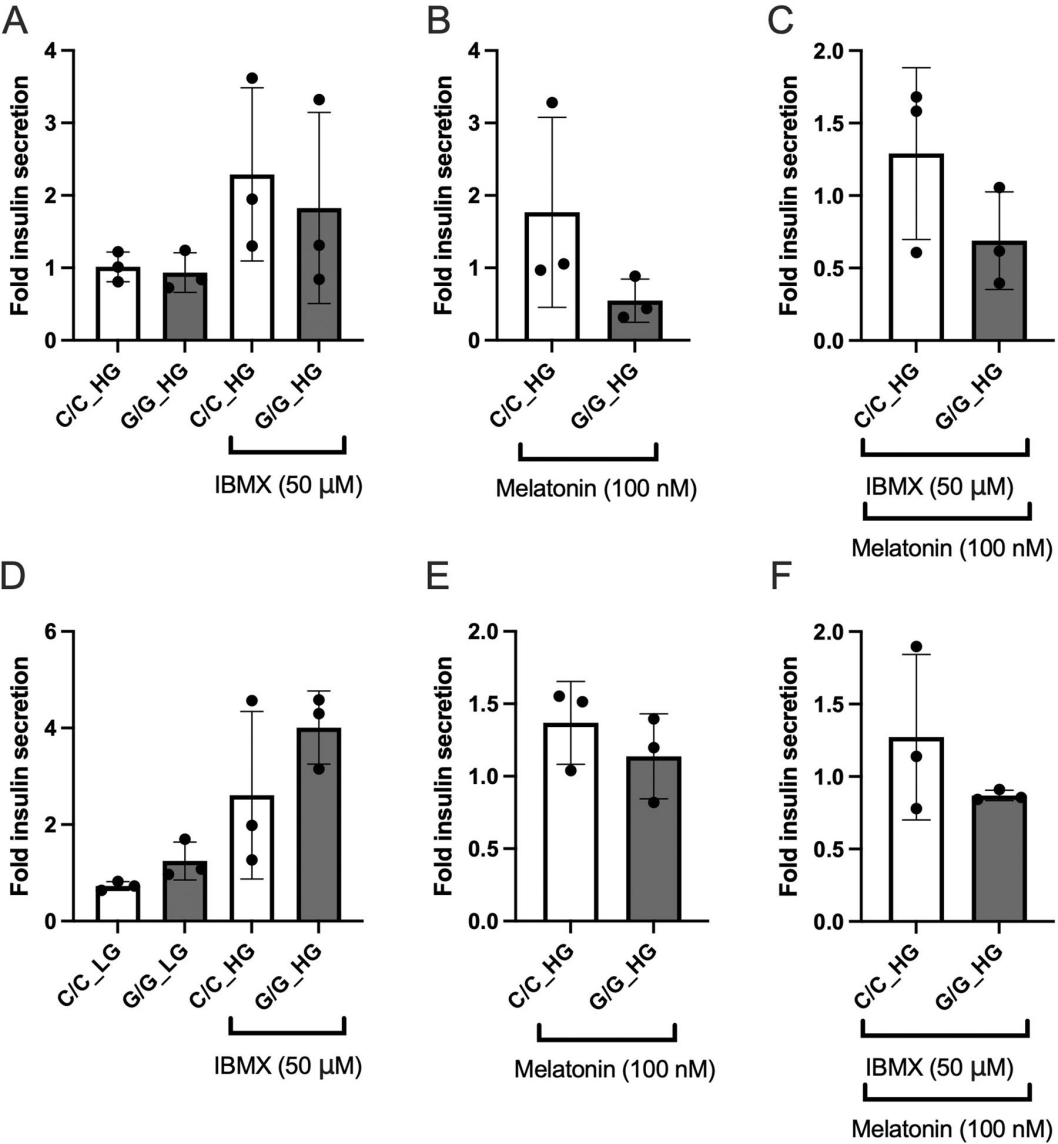
Insulin secretion in differentiated cells. Fold secreted insulin (normalized to low glucose) in hiPSC derived β‐cells (A–C). Differences in fold (1 mM glucose (LG), 20 mM glucose (HG)) and HG + IBMX (50 μM) (A), stimulation with HG and melatonin (100 nM) (B). Stimulation with the combination of HG + IBMX (50 μM) + melatonin (100 nM) (C). Fold secreted insulin (normalized to low glucose) in hESC derived β‐cells (D–F). Differences in fold (1 mM glucose (LG), 20 mM glucose [HG]) and HG + IBMX (50 μM) (D), stimulation with HG and melatonin (100 nM) (E). Stimulation with the combination of HG + IBMX (50 μM) + melatonin (100 nM) (F). (*N* = 3 biological replicates, mean ± SD).

To determine functional changes in isogenic cell lines upon editing of the *MTNR1B* G‐risk allele, we exposed β‐cells (derived from both hiPSC and hESC) to either low (LG 1 mM) or high (HG 20 mM) glucose, and 3‐isobutyl‐1‐methylxanthine (IBMX 50 μM) with or without melatonin (100 nM) (Figure 6A–C; hiPSC derived β‐cells. D–F; hESC derived β‐cells.). Data are presented as fold secretion of glucose control (low glucose [LG]). Here, β‐cells derived from hiPSCs showed no response to glucose stimulation, while a robust stimulation was observed upon the addition of 50 μM IBMX (Figure 6A). These observations align with what most studies of such cells in vitro have reported [19, 20]. Similarly, no effects of high glucose were observed in hESC‐derived β‐cells, but a nominally increased secretory response occurred when cells were subjected to high glucose and IBMX (Figure 6D). The combination of high glucose and 100 nM melatonin nominally reduced insulin release in both hiPSC and hESC derived β‐cells (Figure 6B,E), where carriers of the G/G genotype appeared to be more sensitive to melatonin treatment (e.g., reduced secretion as compared to C‐allele carriers). Lastly, treatment with high glucose, IBMX and melatonin resulted in a nominal reduction in insulin release (Figure 6C,F). Again, this reduction appeared to be more prominent in β‐cells harboring the G/G risk as compared to the C/C nonrisk genotype. In summary, our secretion data suggest that β‐like cells derived from both hiPSCs and hESCs display a limited responsiveness to elevated glucose concentrations. Insulin secretion, however, is enhanced in the presence of IBMX, with no clear evidence for genotype‐dependent differences. However, there is an indication that β‐like cells carrying the G/G risk allele are more sensitive to the well‐known inhibitory effect of melatonin on insulin secretion; this observation requires further validation in larger datasets.

## 4 Discussion

Molecular mechanisms linked to genetic signals identified by GWAS could help elucidate the causes of β‐cell dysfunction in T2D. However, functional consequences of these signals in β‐cells remain unknown. Here, we describe a stem cell‐based approach, utilizing genome editing of hiPSC and hESCs followed by differentiation into β‐cells, with the aim to elucidate functional effects of risk alleles. To this end, we targeted the SNP rs10830963 mapping to an intron of the gene encoding the MTNR1B receptor, which is a robust risk allele for T2D [2, 6, 7, 8, 9].

We performed differentiation of hiPSC and hESC into β‐cells, utilizing a previously established 2D‐based protocol [16]. Both hiPSCs and hESCs differentiated accordingly, with the expected rising expression of endocrine markers, followed by increasing expression of *INS* and *GCG* at mRNA and protein levels during the later stages of differentiation, with *INS* expression being several fold higher at the termination of the differentiation in both hiPSCs and hESCs.

To allow disease modeling, we utilized CRISPR/Cas9 to perform single‐base genome editing of hiPSCs and hESCs. In hiPSCs, we edited the risk allele (G) to nonrisk (C) to generate isogenic cell lines, with genotypes G/G and C/C. Utilizing isogenic cell lines for comparison is essential as genetic heterogeneity between humans is considerable. Ideally, isogenic cell lines will differ only with regard to the edited SNP (from G to C). In addition, hESCs cells, which are heterozygous (C/G) at the rs10830963 locus, were edited to either the nonrisk (C/C) or risk (G/G) genotype. High efficiency ( > 90%) and fidelity (minimal indels) were achieved for all cell clones. Previous studies have shown limitations of the CRISPR/Cas9 system in its applicability due to, for example, low transfection efficiency, toxicity, difficulty in obtaining pure clones of edited cells, and off‐target effects [21]. However, here, no issues with editing were encountered.

We have previously shown that the *MTNR1B* rs10830963 SNP is an eQTL in human islets of Langerhans associated with increased *MTNR1B* mRNA levels [2]. To achieve this, evidence suggests that the SNP may influence *MTNR1B* gene expression via increased FOXA2‐bound enhancer activity in islet‐ and liver‐derived cells [14]. In addition to this, allele‐specific differences of the transcription factor NEUROD1 binding in islet‐derived cells have been observed [14]. This notwithstanding, demonstrating cellular or molecular proof of function of rs10830963 is critical, as it could merely act as a tag SNP in the haploblock, where additional SNPs are present, and may influence gene expression and cellular function, either separately or combined. Presently, causality of rs10830963 can only be determined by specifically editing this SNP in a relevant model and determine functional consequences thereof (i.e., prove that the SNP is an eQTL that increases *MTNR1B* expression in carriers of the G allele). Indeed, this may prove to be difficult, as the effect size of a single SNP may be relatively small, given that a plethora of genetic signals ( > 1000) associate with glycaemic traits and insulin secretion in T2D [4, 5].

To this end, mRNA expression of *MTNR1B* in risk (G/G) and nonrisk isogenic clones (both hiPSCs and hESCs) was determined. Overall, expression of *MTNR1B* was very low, both in sorted and unsorted β‐cells. This is a limitation of our study, as we are investigating potential functional effects of an eQTL. The reasons accounting for this low level of *MTNR1B* expression may be manyfold. One major reason for low *MTNR1B* expression may be inherent in our model—(e.g., stem cell derived β‐cells). Difficulties to detect and determine the expression of *MTNR1B* mRNA and protein in islet cells have previously been extensively discussed: studies utilizing global knock out mice of *Mt1* and *Mt2* reveal islet immunostaining of Mt2 protein localized to β‐cells, while Mt1 protein was observed in α‐cells [22]. *MTNR1A* and *MTNR1B* protein expression and abundance in human pancreatic β‐cells have been confirmed [2, 23, 24], but with lower *MTNR1A* and *MTNR1B* expression in α‐cells [24]. Others show *MTNR1A* mRNA expression predominantly in α‐cells of human islets, while *MTNR1B* expression were virtually undetectable [25]. The inherently low mRNA expression of *MTNR1B* in human islets has made it difficult to determine islet cell‐specific localization, even with recent efforts using single cell RNA sequencing [26]. In this context, studies on islet cell heterogeneity, genetic variation, metabolic state, half‐life and stability of the *MTNR1B* protein/mRNA may provide further insight. Interestingly, *MTNR1B* expression was readily detected in isolated human islets from both risk and nonrisk allele carriers, with a significant increase in expression in risk allele carriers [2], using qPCR or RNA sequencing [6]. Importantly, there is ample evidence that the common *MTNR1B* variants are associated with an increased risk of T2D [2, 6, 27, 28, 29, 30, 31]. However, low expression levels of *MTNR1B* mRNA in human pancreatic β‐cells have raised questions about the physiological relevance of melatonin signaling in these cells. Another suggestion is that timing of food intake to when melatonin levels are elevated (i.e., night) may impair glucose metabolism, particularly in *MTNR1B* G allele carriers [32], potentially via centrally mediated effects. Indeed, such an explanation could provide a mechanistic explanation for how circadian misalignment and genetic risk interact to increase T2D risk. It also underscores the metabolic importance of meal timing [33]. Disruption of both central and peripheral circadian rhythms—potentially due to rare, loss‐of‐function variants—may also contribute to the development of metabolic disorders, including T2D [34]. While several studies have consistently linked the common *MTNR1B* variant to metabolic traits, the association of rare, loss‐of‐function *MTNR1B* variants with T2D has also been shown [34, 35].

Of note here, we detected MTNR1B protein both in undifferentiated hiPSCs and hESCs and in differentiated β‐cells with Western blot and immunocytochemistry. These experiments were further validated with Western blot experiments in *MTNR1B* knockout hiPSCs, with results confirming significantly reduced abundance of MTNR1B protein. We detected MTNR1B protein in both undifferentiated hiPSCs and hESCs, as well as in differentiated β‐like cells, using Western blot and immunocytochemistry. To further assess the specificity of these signals, we performed additional Western blot analyses of hiPSCs in which the *MTNR1B* allele had been deleted by CRISPR/Cas‐editing: this revealed reduced abundance of MTNR1B protein reflecting the efficiency of *MNTR1B* knock out (45% vs. 26%; WB signal and knock out efficiency, respectively). Interestingly, the level of MTNR1B protein expression was nominally higher in β‐like cells derived from risk allele carriers, although this difference did not reach statistical significance (*p* = 0.09). This trend is consistent with the interpretation that the *MTNR1B* risk SNP (rs10830963) functions as an eQTL, under the assumption that increased mRNA levels are accompanied by a corresponding increase in protein levels. Given the modest sample size and the borderline statistical support, these observations should be interpreted with caution until confirmed in larger datasets from independent experiments.

Importantly, the effect size of a single SNP alteration may be obscured by numerous other genetic signals. Indeed, studies similar to ours, but of monogenic forms of diabetes (e.g., MODY), report functional changes. However, in these cases the effect size is profoundly greater [36, 37, 38]. This notwithstanding, linking common variants to disease mechanisms has major translational potential.

Consistent with other studies [19, 20], our stem cell derived β‐cells do not respond robustly in vitro to exogenously added glucose with increased insulin release. This notwithstanding, we observed that the addition of IBMX led to an increase in insulin release, with similar responses regardless of genotype. Under high‐glucose conditions, as well as in the presence of IBMX, melatonin nominally and modestly reduced insulin secretion in β‐cells derived from both hiPSCs and hESCs. This inhibitory effect appeared somewhat more pronounced in β‐like cells carrying the G/G genotype. These findings raise the possibility that β‐like cells carrying the G‐allele may exhibit a greater sensitivity to the inhibitory action of melatonin. If substantiated, such an effect would be consistent with the hypothesis that the rs10830963 SNP at the *MTNR1B* locus mediates an inhibition of insulin release, potentially explaining the diabetogenic influence of the G‐allele. However, given the preliminary nature of these data, derived from time‐consuming and labor‐intensive stem cell experiments, further and larger independent experiments are required to confirm this interpretation.

Importantly, melatonin has previously been shown to decrease insulin secretion, where acute effects of melatonin on insulin release are mediated by reduced formation of cAMP with a net inhibitory effect on insulin release [11, 12, 39]. Stimulation of β‐cells by glucose is known to increase intracellular cAMP, whereas melatonin blocks cAMP formation using various signaling pathways in clonal β‐cells, rodent and human islets [11, 12, 40, 41, 42]. Of note, stimulatory effects of melatonin on insulin secretion under certain conditions have also been reported, particularly following prolonged exposure in rodent islets [42] and human islets [25]. Besides the well‐known receptor mediated effects, melatonin can act as an antioxidant and reportedly reduces oxidative stress in β‐cells in vitro [43, 44].

It is established that stem cell derived β‐cells mature and function better in vivo, following transplantation into mice [20], while physiological responses in vitro are less robust. The reduced insulin secretory response of β‐cells derived from stem cells likely emanates from lack of humoral factors, and/or formation of a permissive niche, as well as of microvasculature, all of which are present in vivo during organogenesis, thereby promoting β‐cell maturation and function. Recently, however, several new protocols have been developed with improved yield and functionality of hiPSC‐derived β‐cells, utilizing 3D‐based approaches [45, 46, 47, 48]. Still, a certain degree of immaturity appears to be present in all stem‐cell derived β‐cells [20]. One reason for this was recently explored by metabolic profiling of stem cell derived β‐cells to determine their capacity to sense glucose [49]. Here, reduced anaplerotic cycling in the mitochondria was identified as a potential cause of reduced glucose‐stimulated insulin secretion. Importantly, insulin secretion could be recovered by challenging stem cell‐derived β‐cells with intermediate metabolites from the TCA cycle and late (but not early) glycolysis; this resulted in robust, bi‐phasic insulin release in vitro similar in magnitude to functionally mature human islets [49]. Another reason for the inability of hESC‐ and hiPSC‐derived β‐cells to respond to glucose may be that our and similar differentiation protocols yield polyhormonal cells (e.g., INS^+^ and GCG^+^ cells). Bruin et al., [50] have comprehensively characterized hESC‐derived bihormonal cells: they show that these cells are responsive to KCl and arginine, but not glucose, in perifusion studies. An explanation for loss of glucose sensing in these cells could be lack of GLUT1 protein and reduced K_ATP_‐channel activity. Indeed, they report that the expression of the SUR1 subunit of the K_ATP_‐channel was ~ fivefold lower than KIR6.2. Combined, this suggests that an impaired ratio of SUR1:KIR6.2 may contribute to the observed K_ATP_‐channel defects in hESC‐derived islet endocrine cells, along with lack of GLUT1, which may underlie the absence of glucose‐stimulated insulin secretion. Indeed, we observed expression of *GCG* in our hESC‐ and hiPS‐derived β‐cells, albeit at several fold lower levels than that of *INS*.

It is clear that utilizing hiPSC as cellular systems to model disease mechanisms will become increasingly useful in biomedical research [51], including T2D research, where hiPSCs derived from humans could serve as versatile tools to understand disease mechanisms. There is strong evidence that the common *MNTR1B* variant (rs10830963) is associated with increased T2D risk [2, 6, 27, 28, 29, 30, 31, 52], and perturbed β‐cell function. Functional studies to determine the precise molecular mechanisms linking the *MTNR1B* variants to β‐cell dysfunction are therefore required. Our approach to understand the molecular underpinnings of the pathogenetic effects of the *MTNR1B* risk SNP was to evaluate functional outcomes in isogenic hiPSCs and hESCs carrying risk and nonrisk alleles. Our successful single‐base gene editing approach by CRISPR/Cas9 enabled functional studies of the *MTNR1B* risk allele. However, differentiation protocols need to be improved and further developed to increase maturity and yield of β‐cells. Here, 3D‐based protocols hold promise to generate islet cell‐like clusters, resembling primary tissue (human islets of Langerhans), to determine direct causality of T2D risk alleles on β‐cell function. Another limitation is that, at this point, the protocols for differentiation into β‐cells are extremely labor‐intensive: this restricts the number of cell preparations that can be generated for functional and expression studies, underpowering the experiments. This is a particular concern when T2D risk alleles are examined and which presumably possess small effect sizes.

## Author Contributions

Study design and conceptualization was by M.F. and H.M., where P.W.F. supported this process. Experiments were performed by T.S., S.K., J.P.M.C.M.C., S.H., F.R., S.G., and A.S. K.Y.F. and O.E. biopsied patients. A.M. and H.S. guided the development of the differentiation protocol. A.R. manages the DIACT study and performed G.W.A.S. and genotyping of patients in the study. M.F. wrote the manuscript together with T.S., S.K., S.H. and F.R. All authors have read and approved the manuscript prior to submission.

## Conflicts of Interest

The authors declare no conflicts of interest.

## Supporting information


**Supporting Figure 1:** Selected hiPSCs and hESCs are pluripotent. hiPSCs generated from fibroblasts (A‐D) and hESCs cell (HUES4) line (E‐G) show robust protein expression of pluripotency markers OCT4 (green) and TRA‐1‐81 (red). Scale bar represents 100 μm, and the images were captured at 10X magnification with a confocal microscope. **Supporting Figure 2:** MTNR1B protein is expressed in pluripotent stem cell lines. hiPSCs (A‐D) and hESCs (E‐G) show clear protein expression of MTNR1B (green). The scale bar represents 100 μm, and the images were captured at 10X magnification with a confocal microscope. **Supporting Figure 3:** Tile scale images, immunocytochemistry and mRNA expression. hiPSCs (A, B) and hESC (C) ssuccessfully produced C‐peptide (green) cells. Distribution of these cells can be observed over the entire surface area of a well. Images are captured at 10X magnification with a confocal microscope. Representative mages of C‐peptide positive hiPSC derived β‐cells (D), glucagon positive (E), and a merge (F). *GCG* gene expression in hiPSC derived (G) and hESCs derived (H) endocrine cells over the course of differentiation. The scale bar represents 100 μm, and the images were captured at 10X magnification with a confocal microscope. **Supporting Figure 4:** pLenti‐HIP‐GFP virus testing and titrations on clonal EndoC‐βH1 line. Five days post lentivirus infection, cells showed robust expression of GFP (A‐F). The scale bar represents 200 μm, and the images were captured at 10X magnification with a phase contrast microscope. Percentage of GFP^+^ cells (bottom right corner) were assessed via flow cytometry and are presented as scatter plots (G‐L). From 1X to 4X dilution, more than 90% of cells expressed GFP controlled by the human insulin promoter (HIP) (G‐I). **Supplementary Figure 5:** Sorting gating strategy for GFP^+^ (β‐cells) from fully differentiated cells. hiPSCs and hESCs produced GFP^+^ β‐cells at the end of differentiation after GFP‐lentivirus infection (A‐C); cells were selectively sorted from gate p4 (B”’, C”’) using FACS for downstream gene expression analysis. All panels show gating strategy in series to select cells based on (A‐C) size‐granularity (SSC‐A vs FSC‐A), and single cells (FSC‐H vs FSC‐A; SSC‐H vs SSC‐A). (A‐A”’) Panel A shows gating strategy on unstained (negative) control for GFP^+^ gate (p4) positioning. **Supplementary Figure 6:** Western blot of hiPSC MTNR1B knock out and isogenic control cells. Protein detection by Western blot yielded a 37 kD band, corresponding to the MTNR1B protein (upper panel – lane 1‐3 control and 4‐6 knockout) and β‐TUBUBULIN (50 kD) lower panel (A). Quantification, revealed reduced presence of MTNR1B protein in knockout cells as compared to controls (B) (N = 3 biological replicates, mean ± SD) *p* < 0.05. CRISPR‐Cas9‐mediated MTNR1B knockout efficiency using Sanger sequencing. Genomic sequence flanking the guide RNA target site was PCR‐amplified and Sanger‐seqeuenced. The analysis shows 26% knockout‐generating indel mutations ( + 1 and ‐1 base pair) in the cell population after transfection with the denoted guide target. The dotted lines show the CRISPR‐Cas9 cut site. The wild‐type sequence is shown on top of the sequence stack, denoted by + (C). Sanger sequence raw data of the knockout sample (top) and the wild‐type control sample (bottom). The underlined sequence is the target of the guide RNA. Red dotted line denotes the PAM site. Dashed vertical lines denote the cut site (D). Uncut original blots of MTNR1B (E) and β‐TUBULIN (F), combined with image of ladder (G). **Supplementary Table 1:** Antibodies used for immunocytochemistry. **Supplementary Table 2:** Taqman assays used for qRT‐PCR.

## Data Availability

The data presented in this article will be made available upon reasonable request.
