# Supplementary material for: Modeling Genetic Risk of β‐Cell Dysfunction in Human Induced Pluripotent Stem Cells From Patients Carrying the MTNR1B Risk Variant
Source: J Pineal Res. 2025 Sep 2;77(5):e70073. doi: 10.1111/jpi.70073 (PMC12405741; doi:10.1111/jpi.70073)

Supplementary figures and tables

Supplementary Table 1. Antibodies used for immunocytochemistry

	Antibody	Catalogue number	Source	Dilution
1	Rabbit anti-OCT4	PA5-27438	Invitrogen	1 in 500
2	Mouse anti-TRA-1-81	MA1-024	Invitrogen	1 in 500
3	Rabbit anti-MTNR1B	PA5-102107	Invitrogen	1 in 500
4	Rat anti-cPeptide	GN-1D4-s	DSHB*	1 in 20
5	Goat anti-PDX1	ab47383	Abcam	1 in 5000
6	Rat anti-NKX6.1	F55A10	DSHB	1 in 500

- https://dshb.biology.uiowa.edu

Supplementary Table 2. Taqman assays used for qRT-PCR

	TacMan Assays	Catalogue number	Source
1	*TBP* (housekeeping)	Hs00427620_m1	ThermoFisher
2	*PPIA* (housekeeping)	Hs04194521_s1	ThermoFisher
3	*MTNR1B*	Hs00173794_m1	ThermoFisher
4	*INS*	Hs02741908_m1	ThermoFisher
5	*PDX1*	Hs00236830_m1	ThermoFisher

Supplementary figure 1.

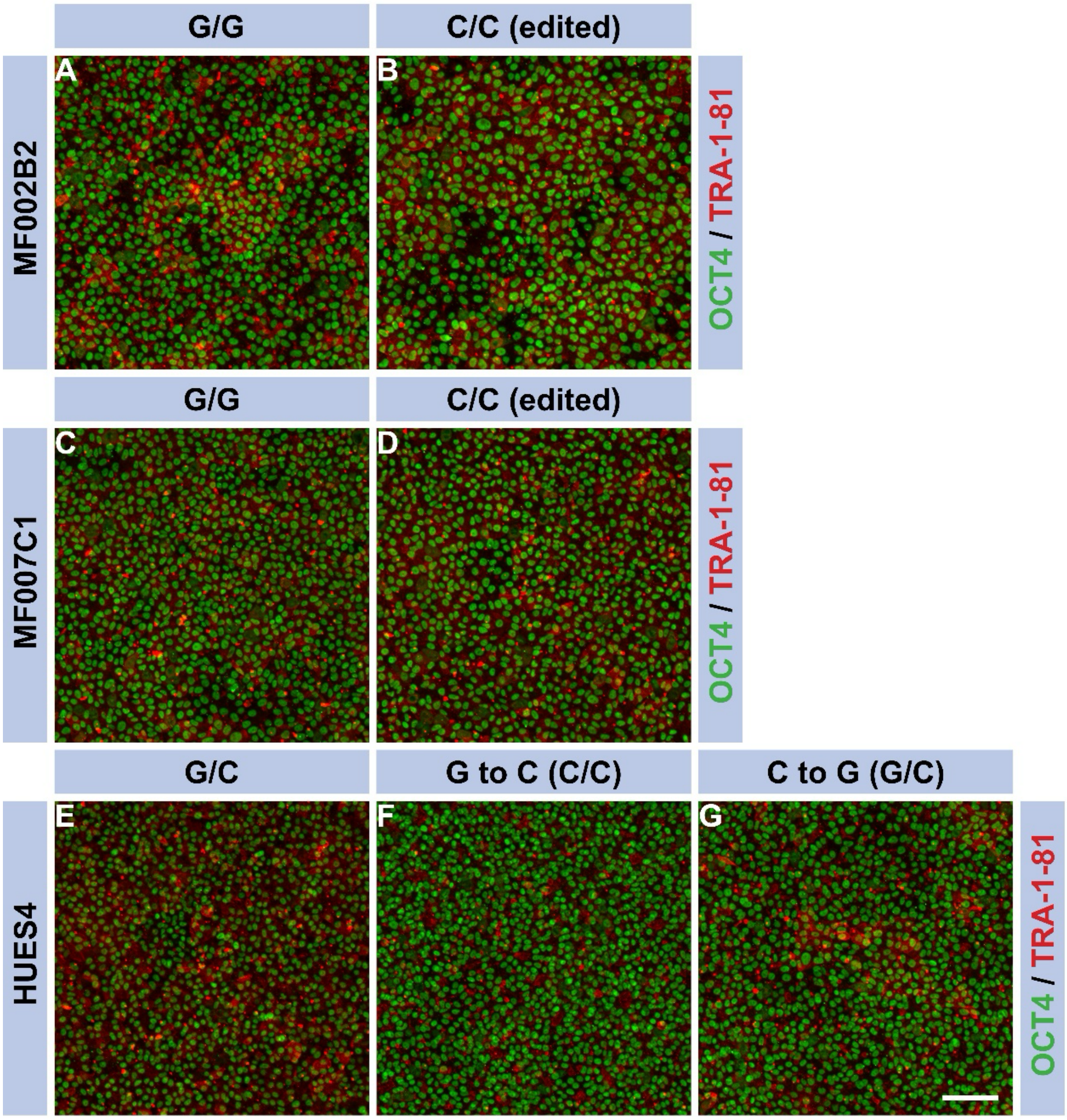

Supplementary figure 2.

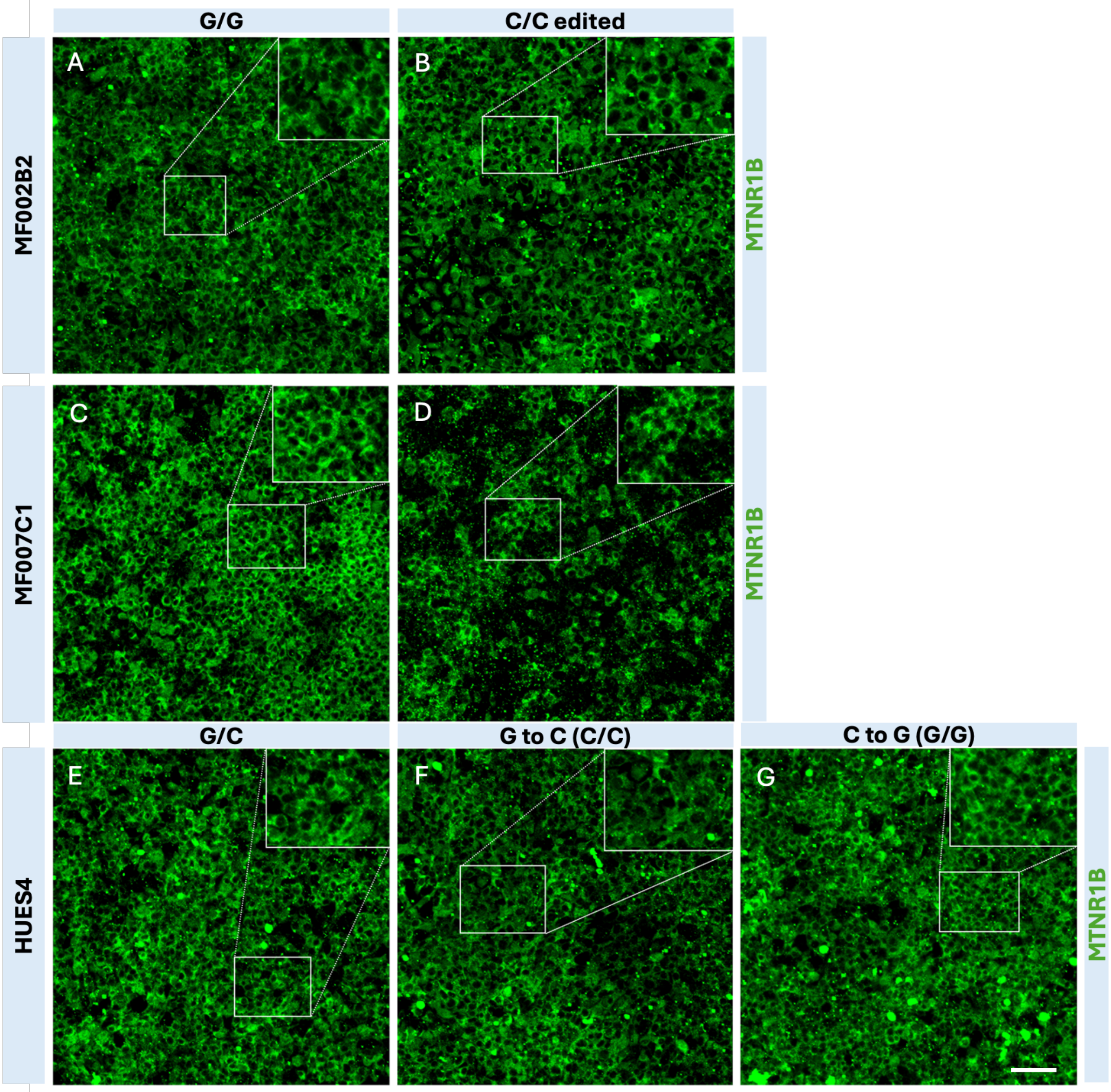

Supplementary figure 3.

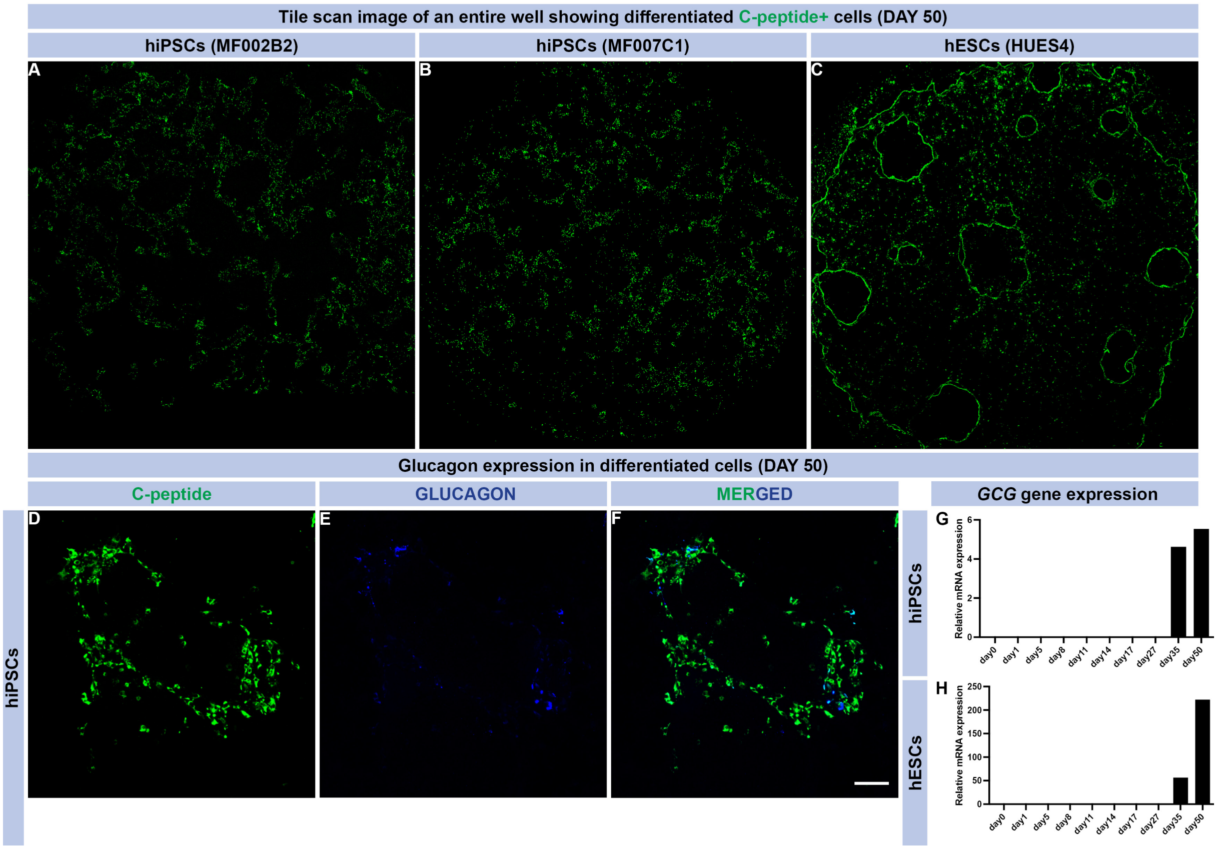

Supplementary figure 4.

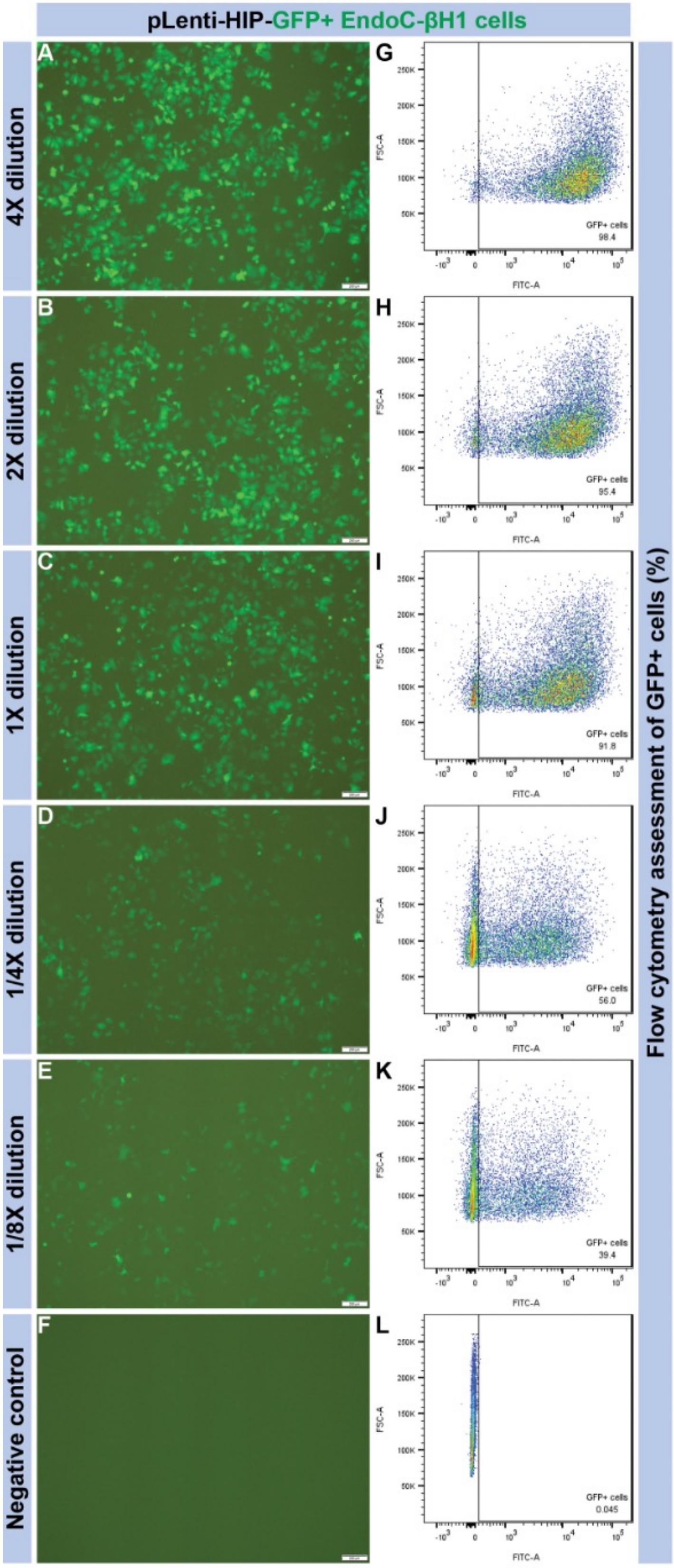

Supplementary figure 5.

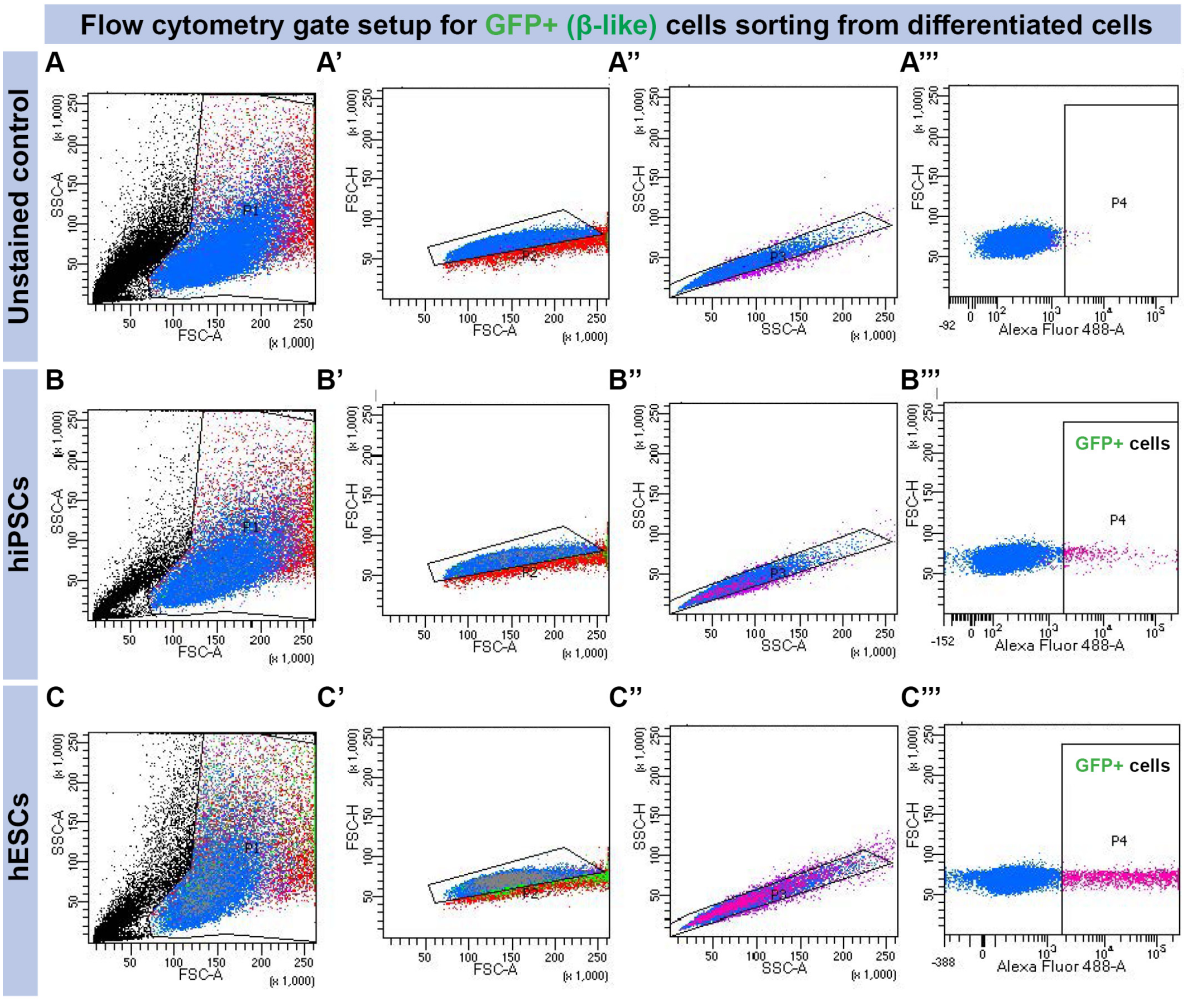

Supplementary Fig 6.

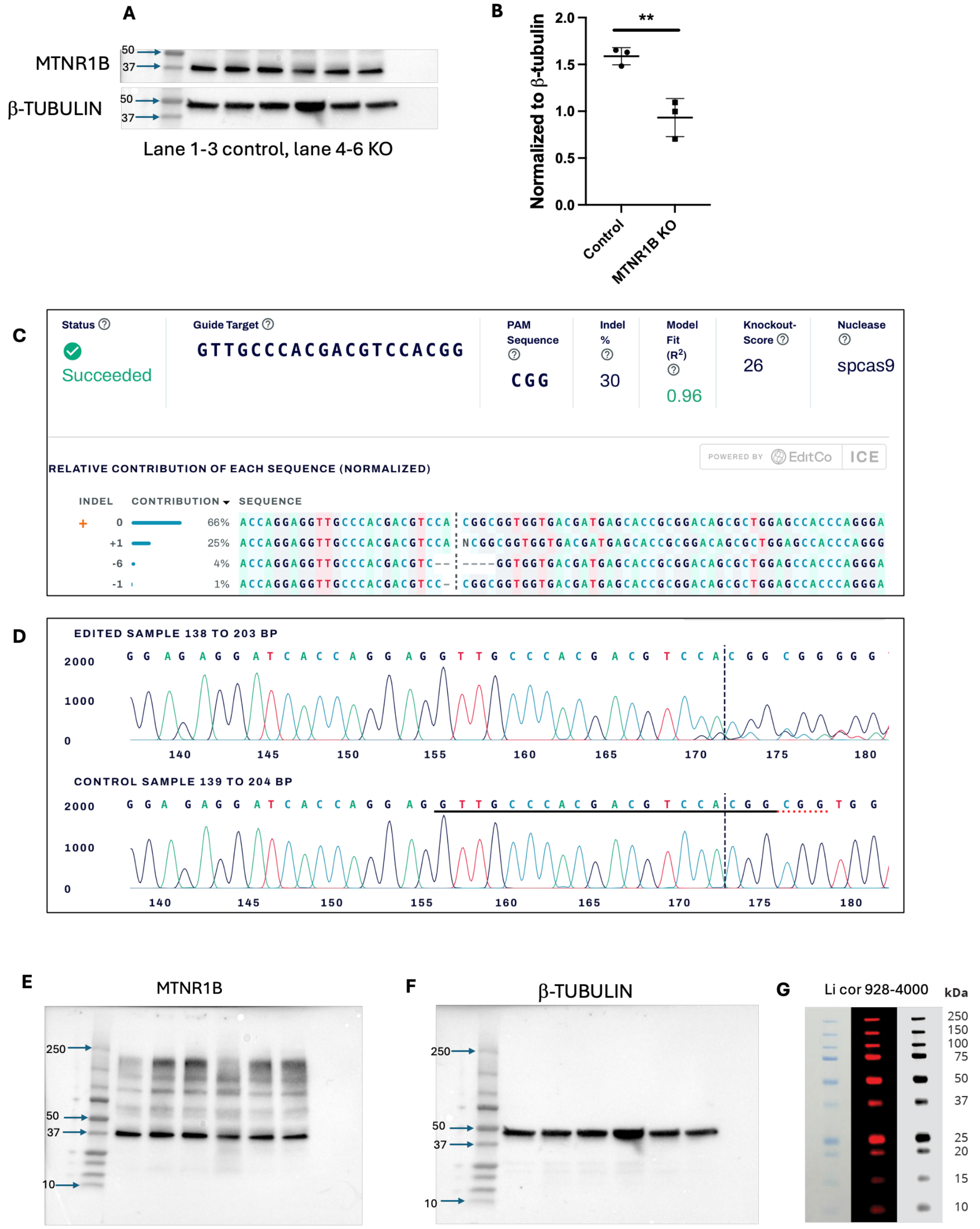

Supplement: Supplementary file 1 — Supporting Figure 1: Selected hiPSCs and hESCs are pluripotent. hiPSCs generated from fibroblasts (A‐D) and hESCs cell (HUES4) line (E‐G) show robust protein expression of pluripotency markers OCT4 (green) and TRA‐1‐81 (red). Scale bar represents 100 μm, and the images were captured at 10X magnification with a confocal microscope. Supporting Figure 2: MTNR1B protein is expressed in pluripotent stem cell lines. hiPSCs (A‐D) and hESCs (E‐G) show clear protein expression of MTNR1B (green). The scale bar represents 100 μm, and the images were captured at 10X magnification with a confocal microscope. Supporting Figure 3: Tile scale images, immunocytochemistry and mRNA expression. hiPSCs (A, B) and hESC (C) ssuccessfully produced C‐peptide (green) cells. Distribution of these cells can be observed over the entire surface area of a well. Images are captured at 10X magnification with a confocal microscope. Representative mages of C‐peptide positive hiPSC derived β‐cells (D), glucagon positive (E), and a merge (F). GCG gene expression in hiPSC derived (G) and hESCs derived (H) endocrine cells over the course of differentiation. The scale bar represents 100 μm, and the images were captured at 10X magnification with a confocal microscope. Supporting Figure 4: pLenti‐HIP‐GFP virus testing and titrations on clonal EndoC‐βH1 line. Five days post lentivirus infection, cells showed robust expression of GFP (A‐F). The scale bar represents 200 μm, and the images were captured at 10X magnification with a phase contrast microscope. Percentage of GFP+ cells (bottom right corner) were assessed via flow cytometry and are presented as scatter plots (G‐L). From 1X to 4X dilution, more than 90% of cells expressed GFP controlled by the human insulin promoter (HIP) (G‐I). Supplementary Figure 5: Sorting gating strategy for GFP+ (β‐cells) from fully differentiated cells. hiPSCs and hESCs produced GFP+ β‐cells at the end of differentiation after GFP‐lentivirus infection (A‐C); cells were selecti [file JPI-77-e70073-s001.pdf]
